# Long-term clinical outcomes of peritoneal dialysis patients: 9-year experience of a single centre in Turkey

**DOI:** 10.3906/sag-1909-98

**Published:** 2020-04-09

**Authors:** Nihan TEKKARIŞMAZ, Dilek TORUN

**Affiliations:** 1 Department of Nephrology, Faculty of Medicine, Başkent University, Adana Turkey

**Keywords:** Peritoneal dialysis, peritonitis, mortality, patient survival, technique survival

## Abstract

**Background/aim:**

The aim of this study was to evaluate the clinical outcomes and identify the predictors of mortality in peritoneal dialysis patients.

**Materials and methods:**

Medical records of all incident peritoneal dialysis (PD) patients followed up between January 2011 and May 2019 were reviewed retrospectively. All patients were followed up until death, renal transplantation, transfer to haemodialysis or the end of the study.

**Results:**

A total of 242 patients were included in the study. The incidence of peritonitis was 0.18 (ranging from 0 to 14.9) episodes per patient year. Death occurred in 28% (n: 68) of cases. Age, diabetes mellitus, malignancy and refractory heart failure were independent risk factors for all-cause mortality according to multivariate analysis. The presence of comorbid disease and diabetes mellitus and patients aged > 65 years were associated with increased risk of mortality and decreased patient survival. Peritonitis history was associated with increased risk of mortality. Between peritonitis and peritonitis-free group, there was no significant difference in Kaplan-Meier curves in terms of patient survival.

**Conclusion:**

This is the first study to define 9-year mortality predictors in PD patients in our centre. Although peritonitis is the most feared complication of PD, our study showed that peritonitis did not reduce patient survival.

## 1. Introduction

The prevalence of end-stage renal disease (ESRD) is increasing in the world [1,2]. Peritoneal dialysis (PD) has been an alternative treatment to hemodialysis (HD) for patients with ESRD since 1976 [3,4]. PD is a home-based treatment with many advantages; preservation of residual renal function (RRF), hemodynamic stability, better quality of life and cost savings [5–8]. Survival rates with PD are better than those with HD after 3 years from initiation [5,8,9]. Despite the advantages of PD in quality of life compared with haemodialysis, the prevalence of PD decreases gradually [10,11]. In our country too, total number of PD patients is gradually decreasing over the years [12]. 

Prevalent PD patients in our country as of the end of 2007 were 5307 patients and this number decreased to 3346 at the end of 2017, according to the Turkish Kidney Registry System Reports [12,13]. The long-term benefits of PD are still controversial [5]. Therefore, this study aimed to report a single-center experience and long-term clinical outcomes of PD over a 9-year period. 

## 2. Materials and methods

### 2.1. Participants 

This study was approved by Baskent University Institutional Review Board (Project no: KA19/196) and supported by Başkent University Research Fund. This was a retrospective cohort study. All patients who were initiated on PD at Başkent University Adana Dr. Turgut Noyan Training and Research Hospital, in Turkey, from January 2011 to May 2019, were included. The patients who were younger than 18 years and the patients with a PD history of less than 3 months were excluded from our analysis. The follow-up of the patients was reviewed until death, renal transplantation, transferred to HD or the end of the study in May 2019. 

### 2.2. Clinical procedures 

A double-cuffed Curl Tenckhoff catheter (ArgyleTM Peritoneal Dialysis Catheter Kit, Curl Catch, 2 Cuff, 62 cm) was inserted in all patients in our centre using the laparoscopy technique by general surgeons. After a break period, patients and their caregivers underwent a standard training program after catheterization. Initiation of PD refers to the time, when the patient started to use the PD solutions effectively to performing 2-litre exchanges 4 times per day. The patients were initiated on manual exchanges using Y-sets and twin-bag systems. PD solutions (Physioneal 1.36, 2.27, 3.86% (glucose), Nutrineal, Extraneal; Baxter Healthcare, USA) were utilized in all of PD patients. Dialysis prescription was changed according to individual requirements determined during the follow-up. Continuous Ambulatory Peritoneal Dialysis (CAPD) or Automated Peritoneal Dialysis (APD), is proposed to patients in our institute according to the patient’s wishes and contraindications for the respective PD modality. 

Patients were asked to collect urine for 24 h from patients with appreciable urine output for peritoneal equilibration test and for RRF calculation. Peritoneal transport characteristic was assessed by peritoneal equilibration test. RRF was calculated using the average of urea and creatinine clearance [14]. Patients were recommended to contact their dialysis nurse and/or nephrology doctor by telephone for any medical support. Patients with suspected peritonitis were advised to come to the hospital. All peritonitis patients were treated according to the International Society for Peritoneal Dialysis Guidelines [15]. 

### 2.3. Definitions

Anuria was defined as the absence of sufficient urine output. The PD withdrawal due to inadequate dialysis, infection or mechanical reasons (except death, renal transplantation, loss to follow-up) were defined as technique failure. [5,6]. PD-related infections were defined as peritonitis, exit site infections, and tunnel infections [16]. Peritonitis was diagnosed according to the International Society for Peritoneal Dialysis Guidelines [15], which requires at least 2 of the following: 1. Clinical features consistent with peritonitis, i.e. abdominal pain and/or cloudy dialysate fluid, 2. Dialysate white cell count > 50% polymorphonuclear leukocytes, >100/μL or >0.1 × 109/L (after a waiting time of at least 2 h), 3. Positive dialysate culture. Noninfectious complications such as hernia, dialysate leakage, drainage disorder and ileus were defined as mechanical complications. Technique survival was defined as the time interval between the date of initiation of PD and the date of withdrawal of the catheter (except death, renal transplantation, loss to follow-up). Patient who died for any reason under PD was defined as the mortality group and the remaining patients who continued PD at the end of the study period (30 May 2019) were defined as the survival group (except renal transplantation, transfer to HD, loss to follow-up). Early death was defined as each death occurring in the first 6 months of PD. Patient survival was defined as the probability of survival of patients under PD treatment [8,17]. In the survival analysis, the starting point was defined as the first day of PD. Endpoint of study for each patient was death for any reason, renal transplantation, transfer to HD, loss to follow-up or the end of the study in May 2019.

The patients with peritonitis history were defined as peritonitis group and the rest were defined as nonperitonitis group. Also, as such the patients were categorized as diabetics group, nondiabetics group, comorbidity group and noncomorbidity group, patients were also categorized per age; aged <40 years, aged between 40–65 years and aged >65 years groups. 

The following data were obtained from the patient files.

### 2.4. Baseline demographic and clinical data 

Age, sex, body mass index (BMI), systolic and diastolic blood pressure measurements, aetiology of ESRD (diabetes mellitus, hypertension, glomerulonephritis, tubulointerstitial nephritis, polycystic kidney diseases, obstructive nephropathy, amyloidosis, refractory heart failure), the presence of comorbidities (diabetes mellitus, hypertension, cardiovascular disease, malignancy, tuberculosis, cerebrovascular disease, and chronic lung disease), patients with parathyroidectomy performed, and medication history were recorded. The mean of the last 2 values for levels of haemoglobin, serum creatinine, calcium, phosphorus, albumin, intact parathyroid hormone, transferrin saturation, ferritin, C-reactive protein, total cholesterol, LDL-cholesterol, triglyceride values and hepatitis serology were recorded. Previous HD history, PD modality (CAPD or APD), ultrafiltration volume, use of Icodextrin and Nutrineal dialysate, and peritoneal transport characteristic were recorded. Daily urine volume, Kt/V urea, and RRF of patients at the onset of PD and at the last control or at the 12-month evaluation before their death were recorded.

### 2.5. PD outcomes 

Follow-up duration, dialysis duration, technique failure rate, technique survival, PD-related infections, (Peritonitis episodes, tunnel infection, exit-site of infection), mechanical complications, mortality rate and patient survival were reviewed for all patients at the end of the study. 

### 2.6. Subgroups

Patients were divided into different subgroups as follows; group excluding early deaths, group excluding patients with prior HD history, diabetics vs. nondiabetics group, peritonitis vs. nonperitonitis group, comorbidity group, noncomorbidity group and patients aged <40 years vs. aged between 40–65 years, and aged > 65 years groups. 

### 2.7. Statistical analysis

Statistical analysis was performed using the statistical package SPSS software version 23.0 (IBM Corp., Armonk, NY, USA). For each continuous variable, normality was checked by Kolmogorov–Smirnov and Shapiro–Wilk tests and by histograms. All numerical data are expressed as median values (minimum–maximum) or as proportions. Comparisons between groups were applied using student t test for normally distributed data and Mann–Whitney U test were used for the data not normally distributed. The categorical variables between the groups were analysed by using the chi- square test. Overall survival time was defined as the years elapsed between the first day of PD and death for any reason, renal transplantation, transfer to HD, loss to follow-up or the end of the study in May 2019. Overall survival was analysed using the Wald test, and the log-rank test was used to examine their relationship when different parameters were applied. The survival curve was plotted using the standard Kaplan-Meier methodology. A Cox regression model with stepwise selection was done to identify variables. Thereafter, the treatment effect adjusted for these selected variables was calculated. The Cox model was also used to examine the interaction of treatment effect with subgroup status in an exploratory analysis. Differences in the best overall response rates between the treatment groups were analysed with the Cochran-Mantel-Haenszel test.

## 3. Results

### 3.1. Demographic and clinical results 

A total of 242 patients were included in the study. The mean age of incident patients was 52.5 ± 16.6 years at the onset of PD. Amongst the patients 59 of them (24.4%) were aged >65 years and 55 of them (22.7%) were aged <40 years, and 115 of them (47.5%) were male. Diabetic nephropathy was present in 30.2% of all patients. The PD modality was APD in 31.4% of patients. The patients’ baseline demographic and clinical characteristics, medication history and biochemical parameters, and peritoneal dialysis related data were presented in Table 1, in Table 2, and in Table 3, respectively.

**Table 1:** Baseline demographic and clinical characteristics of the peritoneal dialysis patients.

	Mortality group (n: 68)	Survival group (n: 174)	Total (n: 242)	P
Age (years)**	58.9 ± 15.8	47.6 ± 15.6	52.5 ± 16.6	0.001#
Sex (male)*	49 (46.2)	66 (48.5)	115 (47.5)	0.410
BMI (kg/m2) **	27.8 ± 6.1	27.7 ± 5.3	27.7± 5.7	0.920
Systolic BP (mmHg)**	117.6 ± 22.6	125.8 ± 22.4	122 ± 23	0.006#
Diastolic BP (mmHg)**	69.5 ± 11.1	72.7 ± 10.0	71 ± 11	0.018#
Etiology of ESRD				
Diabetes mellitus*	48 (45.3)	25 (18.4)	73 (30.2)	0.000#
Hypertension*	24 (22.6)	45 (33.1)	69 (28.5)	0.192
Glomerulonephritis*	2 (1.9)	23 (16.9)	25 (10.3)	0.033#
TIN*	9 (8.5)	6 (4.4)	15 (6.2)	0.011#
PCKD*	3 (2.8)	10 (7.4)	13 (5.4)	0.924
Obstructive nephropathy*	1 (0.9)	6 (4.4)	7 (2.9)	0.690
Amyloidosis*	0 (0)	2 (1.5)	2 (0.8)	0.920
Refractory heart failure*	8 (7.5)	2 (1.5)	10 (4.1)	0.000#
Unknown*	9 (8.5)	16 (11.8)	25 (10.3)	0.487
Comorbidity*	93 (87.7)	86 (63.2)	179 (74)	0.000#
Malignancy*	9 (8.5)	2 (1.5)	11 (4.5)	0.000#
Tuberculosis*	4 (3.8)	6 (4.4)	10 (4.1)	0.619
CVD*	4 (3.8)	1 (0.7)	5 (2.1)	0.000#
Parathyroidectomy*	12 (11.3)	12 (8.8)	24 (10)	0.330

BMI: body mass index, BP: blood pressure, ESRD: End-stage renal disease, TIN: Tubulointerstitial nephritis, PCKD: Polycystic kidney diseases, CVD: Cerebrovascular disease, # P < 0.05, *n (%), **mean ± SD

**Table 2:** Patients’ medication histories and biochemical parameters.

	Mortality group (n: 68)	Survival group (n: 174)	Total (n:242)	P
Medication history				
Erythropoietin*	66 (62.3)	92 (67.6)	158 (65.3)	0.230
Iron*	70 (60.6)	104(76.5)	174 (66)	0.100
Vitamin D*	92 (53.8)	57 (67.6)	149 (61.6)	0.090
Biochemical parameters				
Hb (g/dL) **	10.5 ± 1.7	10.5 ± 1.4	10.5 ± 1.5	0.732
Cr (mg/dL) **	7.8 ± 2.4	9 ± 3.4	8.5 ± 3.1	0.005#
Ca (mg/dL)**	8.46 ± 0.82	8.55 ± 0.82	8.51 ± 0.82	0.389
P (mg/dL) **	4.63 ± 1.23	4.85 ± 1.21	4.75 ± 1.22	0.170
Alb (g/L)**	3.18 ± 0.45	3.52 ± 0.41	3.3 ± 0.4	0.001#
PTH (pg/mL)**	469 ± 396	495 ± 434	484 ± 417	0.621
T. Sat %, **	28 ± 18	32 ± 16	30 ± 17	0.091
Fer, ng/mL**	667 ± 592	556 ± 519	605 ± 554	0.124
CRP, mg/L**	34 ± 41	19 ± 30	26 ± 36	0.000#
TC, mg/dL**	186 ± 47	193 ± 42	190 ± 45	0.093
LDL, mg/dL**	114 ± 42	114 ± 38	114 ± 40	0.621
TG, mg/dL**	194 ± 125	193 ± 116	194 ± 120	0.898
Hepatitis B*	2 (1.9)	4 (2.9)	6 (2.5)	0.465
Hepatitis C*	6 (5.7)	5 (3.7)	11 (4.5)	0.330

Hb: Haemoglobin, Cr: Creatinine, Ca: Calcium, P: Phosphorus, Alb: Albumin, PTH: Parathyroid hormone, T. Sat: Transferrin saturation, Fer: Ferritin, CRP: C-Reactive protein, TC: Total cholesterol, LDL: Low density lipoprotein, TG: Triglyceride, # P < 0.05, *n (%),** mean ± SD

**Table 3:** Patients’ peritoneal dialysis related data.

	Mortality group(n: 68)	Survival group(n: 174)	Total(n: 242)	P
Previous HD history*	23 (21.7)	23 (16.9)	46 (19)	0.410
Automatic PD*	27 (25.5)	49 (36)	76 (31.4)	0.090
Anuria*	39 (57.4)	64 (47.1)	103 (50.5)	0.180
Urine V (mL)***	835 (325–2285)	845 (222–2650)	835 (222–2650)	0.810
Ultrafiltration V (mL)***	1500 (500–3500)	1500 (300–2800)	1500 (300–3500)	0.825
Icodextrin*	105 (94.1)	128 (99.1)	233 (96.3)	0.082
Nutrineal*	55 (52)	61 (45)	116 (47.9)	0.301
Low permeability* †	10 (9.4)	13 (9.6)	23 (9.5)	0.076
Kt/V urea**	2.03 ± 0.49	2.34 ± 0.74	2.24 ± 0.69	0.002#
RRF (mL/min/1.73 m2) ***	4 (2-6.2)	2.7 (0.2-16)	2.7 (0.2-16)	0.35
Technique survival (years)***	4.1 (0.6–18.3)	3.2 (0.1–22.3)	4.6 (0.1–22.3)	0.075
Peritonitis history*	77 (72.6)	65 (47.8)	142 (58.7)	0.003#
Tunnel infection*	5 (4.7)	4 (2.9)	9 (3.7)	0.348
Exit-site infection*	0 (0)	5 (3.7)	5 (2.1)	0.054

HD: Haemodialysis, PD: Peritoneal dialysis, V: Volume, Kt/V: Dialysis adequacy, RRF: Residual renal function, † The data for 44 patients (18.2%) were missing, # P < 0.05, *n (%), ** mean ± SD, *** median (range).

### 3.2. PD outcomes 

Total number of our PD patients was 78 at the starting date of the study. At the end of the study; a total of 242 patients underwent PD. Previous HD history were present in 19% (n: 46) of the patients, and the mean period of HD therapy prior to PD was 1 ± 2.9 years. Total follow-up period was 23.958 patient months (2.178 patient years). The mean dialysis duration was 4.3 ± 3.9 patient-years. During the follow-up period, only 69 patients stayed on PD, 68 patients died. Patients (22 of them) received renal transplantation, 75 patients were permanently transferred to HD, and 8 patients were lost to follow-up (Table 4).

**Table 4:** Long-term results of peritoneal dialysis patients.

PD follow-up time (years)**	4.3 ± 3.9
Mortality rate*	68 (28.1)
Early mortality rate*	8 (3.3)
Patient survival (years)**	3.8 ± 2.9
Technical failure rate*	64 (26.4)
Technical survival (years)***	4.6 (0.1–22.3)
Drop-out causes in PD patients (n: 75)	
PD-related infections*	41 (54.6)
Mechanical complications*	23 (30.6)
Patients preference*	2 (2.6)
Unknown*	9 (12)
Final status of the patients	
Stayed on PD*	69 (28.5)
Died during the study*	68 (28)
Received renal transplantation*	22 (9)
Transferred to haemodialysis*	75 (31)
Loss to follow-up*	8 (3.3)
The causes of death	
Unknown*	22 (32.3)
Infection*	20 (29.4)
Cardiovascular disease*	15 (22)
Malignancy*	5 (7.3)
Cerebrovascular disease*	5 (7.3)
Others*	1 (1.4)

PD: Peritoneal dialysis, *n (%), ** mean ± SD, *** median (range).

### 3.3. Technique failure

PD catheter was removed from 64 patients (26.4%) due to technique failure. Technique failure was not associated with increased risk of mortality (P: 0.26). The main causes of technique failure were PD-related infections (54.6%) and mechanical complications (30.6%). There were 297 episodes of peritonitis that occurred over the total study period. The overall incidence of peritonitis during the 9-year study period was 0.18 (ranging from 0 to 14.9) episodes per patient year which equals 1 peritonitis episode for every 7.3 patient-years or 1 episode of 80.6 patient-months. Mechanical complications were detected in 21.5% (n: 52) of the cases. The most common mechanical complications in our PD patients were obesity, hernia and drainage problem. Complications of PD were given in Table 5. 

**Table 5:** Complications related with peritoneal dialysis.

1. PD-related infections*	41 (16.9)
Peritonitis	
Peritonitis history*	142 (58.7)
Peritonitis episodes (3 or more) *	50 (20.7)
Total number of peritonitis attacks (n)	297
Peritonitis rate (episodes per patients-year)***	0.18 (0–14.9)
Tunnel infection*	9 (3.7)
Exit-site of infection*	5 (2.1)
2. Mechanical complications*	52 (21.4)
Obesity*	79 (32.6)
Hernia*	18 (7.4)
Drainage problem*	17 (7.0)
Ultrafiltration failure*	6 (2.5)
Dialysis failure*	5 (2.5)
Encapsulated sclerosing peritonitis*	3 (1.2)
Dialysate leakage*	2 (0.8)
Ileus*	1 (0.4)

PD: Peritoneal dialysis, *n (%), *** median (range).

### 3.4. Mortality

Of the patients 68 (28%) died, of which 8 (12.5%) were early deaths. Infection was the most common cause of mortality among our PD patients (29.4%) and followed by cardiovascular diseases (Table 4). 

According to univariate analysis; advanced age, low systolic and diastolic blood pressure, the presence of diabetes mellitus, glomerulonephritis, tubulointerstitial nephritis, refractory heart failure, comorbidity, malignancy, and cerebrovascular disease were predictors of mortality (Table 1). Low albumin, Kt/V urea and creatinine levels and high C-reactive protein levels were significantly associated with a higher risk of mortality (Table 2 and Table 3). 

The following 4 clinical variables were selected as independent risk factors for all-cause mortality by multivariate analysis: age (HR, 1.1; 95% CI, 1.04–1.1), diabetes mellitus, (HR, 2.5; 95% CI, 1.4–4.5), malignancy (HR, 3.2; 95% CI, 1.3–7.6), refractory heart failure (HR, 8.3; 95% CI, 2.8–23.9). Advanced age, the presence of diabetes mellitus, malignancy, and refractory heart failure also increased mortality as independent risk factors (Table 6). 

**Table 6:** Multivariate cox regression analysis of patient mortality.

	B	SE	Wald	df	p	HR	95.0% CI for HR		
						Lower	Upper	Age (years)
0.067	0.012	29.72	1	0.0001#	1.1	1.04	1.11	Diabetes mellitus
0.90	0.30	9.14	1	0.02#	2.50	1.40	4.50	Malignancy
1.16	0.44	6.95	1	0.008#	3.2	1.3	7.6	Refractory heart failure
2.11	0.54	15.04	1	0.000#	8.3	2.8	23.9

SE: Standard Error, CI: Confidence interval, HR: Hazard ratio, # P < 0.05.

### 3.5. Technique and patient survival

**Figure 1:**
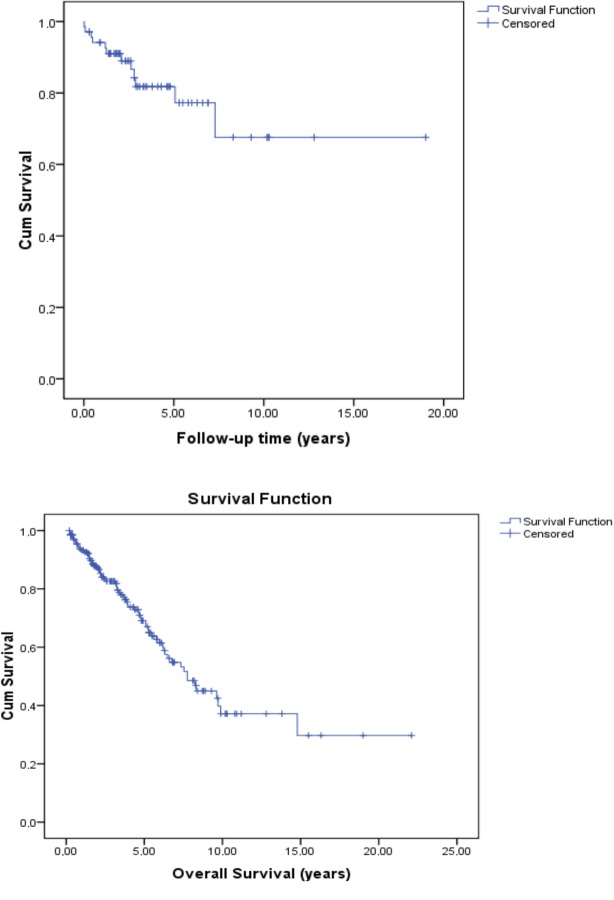
a: Kaplan-Meier curve for overall technique survival of the peritoneal dialysis patients, b: Kaplan-Meier curve for overall survival of peritoneal dialysis patients

The technique survival rates were 92.6%, 89%, 81.8%, 77.3%, and 67.6% at 1, 2, 3, 5 and 9 years, respectively (Figure 1a). The mean technique survival was 14.1 ± 1.4 years (Table 7). The patient survival rates were 93.2%, 81.6%, 68.1%, and 45% at 1, 3, 5, and 9 years after PD initiation, respectively (Figure 1b). The mean patient survival was 10.8 ± 0.9 years (HR, 10.8 ± 0.9; 95% CI, 8.9–12.7) (Table 7).

**Table 7:** Patient and technique survival analysis, and comparison of survival analysis according to various subgroups.

	EMa	SE	95% CI		1.year†	3.year†	5.year†	9.year†	
			LB	UB					
Patient sur	10.8	0.98	8.9	12.7	93.2	81.6	68.1	45.0
Technique sur	14.1	1.4	11.2	16.8	92.6	81.8	77.3	67.6
Age								
p	<40	19.1	1.5	16.1	22.1	95.0	95.0	95.0	88.2	0.000#
40–65	7.2	0.5	6.3	8.1	97.0	85.4	70.6	34.4		
>65	3.7	0.5	2.7	4. 7	75.6	52.4	24.0	0.48		
Sex									
Female	9.4	1.0	7.4	11.4	93.8	87.1	70.4	40.9	0.702
Male	12.0	1.3	9.5	14.5	91.5	76.4	65.1	44.2	
Peritonitis history									
Yes	10.9	1.2	8.7	13.2	94	81	66	45	0.715
No	7. 9	0.9	6.2	9.5	89	81	68	32	
Diabetes mellitus									
Yes	5.5	0.7	4.1	6.9	88.4	64	36.8	16.4	0.000#
No	12.9	1.3	10.4	15.4	96.3	88.9	79.8	53.0	
Comorbidity									
Yes	7.6	0.6	6.5	8.8	91.9	76.8	59.2	31.3	0.000#
No	12.9	1.2	10.7	15.2	94.4	94.4	86.5	74.0	

Sur: Survival, EM: Estimate mean, SE: Standard error, CI: Confidence interval, LB: Lower bound, UB: Upper bound, †Survivor %, # P < 0.05

### 3.6. Upon data analysis of patients within subgroups

The patient survival rates were 91%, 81%, 65%, and 42% at 1, 3, 5, and 9 years, according to the PD time, respectively (HR, mean 10.9 ± 1 years; 95% CI, 8.9–12.9). The patient survival rates were 96%, 84%, 70%, and 43% at 1, 3, 5, and 9 years, after exclusion of early death, respectively (HR, mean 11.1 ± 1 years; 95% CI, 9.1–13). The patient survival rates were 94%, 83%, 69%, and 41% at 1, 3, 5, and 9 years, after exclusion of patients who have prior HD history, respectively (HR, mean 10.9 ± 1.1 years; 95% CI, 8.7–13.1). 

Peritonitis group consisted 58.7% (n: 142) of all patients. The number of peritonitis episodes were higher in the mortality group compared to the survival group (72.6% vs 47.8%, P: 0.003) (Table 3). According to Kaplan Meier survival analysis, no significant difference was observed in the survival rates and survival time between the peritonitis-free and peritonitis groups (mean 10.9 ± 1.2 vs 7.9 ± 0.9 years, P: 0.715) (Figure 2a). 

**Figure 2a:**
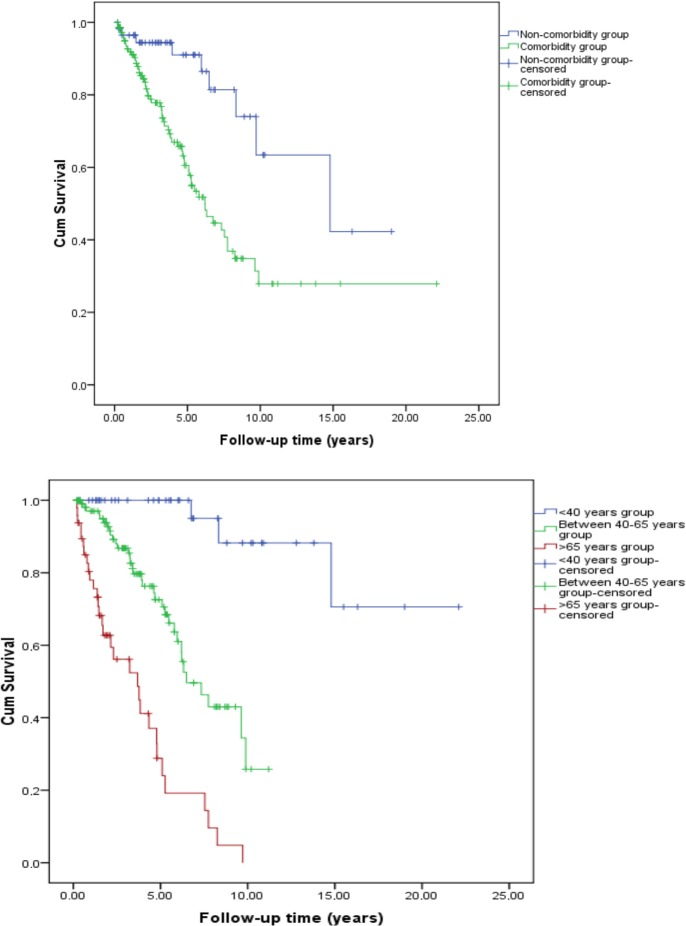
a: Kaplan-Meier curve for survival, peritonitis group vs peritonitis-free group (P = X by log-rank), b: Kaplan-Meier curve for survival, diabetic group vs nondiabetic group, c: Kaplan-Meier curve for survival, comorbidity group vs noncomorbidity group, d: Kaplan-Meier curve for survival, age groups <45 vs between 45–65, vs >65.

**Figure 2b:**
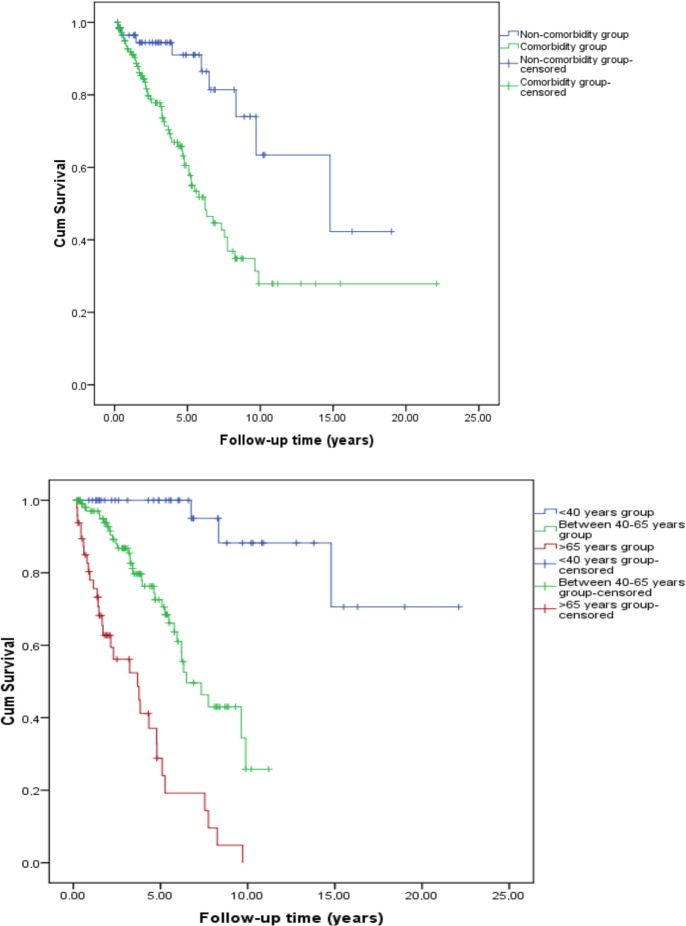
(Continued).

Mortality was higher in diabetic group compared to nondiabetic group (45.3 vs 18.4, P = 0.00) (Table 1). Patient survival time in diabetic group was lower than that of nondiabetic group (mean 5.5 ± 0.7 vs 12.9 ± 1.3 years, P = 0.00) (Table 7). Patient survival rates in diabetic group compared to non-diabetic group were found to be 88.4% vs 96.3%, 64% vs 88.9%, 36.8% vs 79.8%, and 16.4% vs 53% in –1, –3, –5, and –9 years, respectively (Figure 2b).

Mortality was higher in comorbidity group compared to non-comorbidity group (87.7% vs 63.2%, P = 0.00) (Table 1). Patient survival time in comorbidity group was lower than that of non-comorbidity group (mean 7.6 ± 0.6 vs 12.9 ± 1.2 years, P = 0.00) (Table 7). Patient survival rates in comorbidity group compared to noncomorbidity group were found to be 91.9% vs 94.4%, 76.8% vs 94.4%, 59.2% vs 86.5%, 31.3% vs 74% in –1, –3, –5, and –9 years, respectively (Figure 2c).

Mortality was lower in patients younger than 40 years of age and higher in patients older than 65 years of age (21.8% vs 71.1%, P = 0.00). The survival time was lower in patients older than 65 years of age compared to patients younger than 40 years of age (mean 3.7 ± 0.5 vs 19.1 ± 1.5 years, P = 0.00) (Table 7). There was a significant difference in the Kaplan–Meier survival between the different age range: the group aged >65 years had the worst survival rates compared with the 2 groups (aged <40 years and ≥40 years and <65 years) (Figure 2d). 

## 4. Discussion

PD treatment was started in our medical centre for the first time on September 1st of 1998. Over the 9-year period, which this study covers, the total number of patients who commenced renal replacement therapy were 2084 at our hospital. Amongst 2084 patients, 1656 of them underwent HD, 242 of them underwent PD and 186 received renal transplantation. This is the first study to investigate long term outcomes and all-cause of mortality in PD patients in our centre.

The mean age of our patients was similar to that reported in other studies (52.5 vs. 43.9– 62.7 years) [8,18,19]. Unlike other studies, the rate of our elderly patients was higher than other studies (24.4% vs. 10.4%–15.8%) [17,18] and female participants were the majority in our patients (59.3%–63.8% vs 47.5% male) [8,18]. The mean dialysis duration in our patients was longer than other studies (51.6 vs. 27.7–33 months) [8,18]. Compared with other studies, our patients had similar rates of diabetes (24.2%–48.4% vs 30.2%) [4,18,19]. PD offers an alternative treatment option in refractory heart failure and reduces the incidence of decompensation attacks [20]. We had 10 patients (4.1%) who underwent PD due to refractory heart failure. The incidence of tuberculosis in PD patients was reported as 15% in a study from India [9]. In our study, the incidence of tuberculosis was lower (4.1%). Approximately 30%–50% of all PD patients in the worldwide and 22% of all PD patients in our country prefer APD as PD modality [8,12,18,19,21,22]. This rate was similar in our cases. 

Peritonitis was seen as the cause of technique failure in 16.9% of our patients. It was similar to the rates reported in the literature (20.7%–42.6%) [14,19,21]. The prevalence of mechanical complications in our series was lower compared with the different series published in the literature (21.5% vs. 31.2%–36.8%) [4,8]. 

In different studies, the mortality rate of PD patients has been reported to be 10%–28% [3,8,18,23]. Mortality rate of our PD patients at the end of the 9 years period was 28%, in the same range as other studies but on the higher end. The major cause of mortality in our patients was infection (29,4%), followed by cardiovascular diseases (22%). Although rates varied from our results, the same reasons were reported in the other studies. In some studies, cardiovascular diseases were shown to be the most common cause of death (39%–60%), infectious causes were the second most common cause of death (17.6%–40%) among PD patients [8,9,14,17,19,23,24].

The rate of PD survivors was reported to be 58.9% in a 6-year study from Japan [18] and %46.3 in a 2-year study from China [23]. We found the rate of PD survivors to be lower (28.5%), in the 9-year follow-up period. Maybe we can explain this with a longer follow-up period than others. Comparing with the other study patients our rate of transferred to haemodialysis (30.9% vs 8.4%–29.4%) and renal transplantation (9% vs. 1.6%–39%) were similar [10,18,19,23]. 

Just as reported in literature, advanced age was found to be an independent risk factor that increases the risk of mortality [17,18,24,25]. In one study, male sex was reported as a risk factor for mortality, and similar to our study, some studies reported that sex did not play a role in mortality [18,24,25]. Although high BMI was shown to reduce all-cause mortality in one study, no relationship was found between BMI and mortality in our study [26]. One study showed a significant relationship between mortality and previous HD history in PD patients [24]. However, our study did not show any difference. 

Although PD modality was found to be significant in terms of mortality in one study, most studies did not show any difference between CAPD and APD in mortality [17,19,22,24]. In our study, no significant difference was found between 2 groups in terms of mortality. In our study, as in other similar studies, the risk of mortality was significantly higher in patients with diabetes mellitus, cardiovascular disease and comorbidity [17,18,24,25]. 

As also stated in other studies, low serum creatinine level and hypoalbuminemia were found to be risk factors for all-cause mortality in our study [14,17,18,25]. High C-reactive protein was found to be a significant risk factor in our study, as reported in one other study [18]. Although it has been reported in some studies, no correlation was found between phosphorus level and mortality in our study [18,25]. Though some studies have reported that high cholesterol and low HDL are risk factors for mortality in PD patients, there was no correlation between lipid levels and mortality in our patients [18,25].

In our study, anuria and urine volume were not seen as risk factors for mortality. Contrary to our study, the relationship between low urine volume and mortality was reported in previous studies [17]. In one study, low RRF was reported as an independent risk factor that worsened patient survival rates [19]. In our study, RRF was not found as a risk factor for mortality. As also was found in another study, low Kt/V was found to be a risk factor for mortality in our study [25]. The role of peritoneal transport characteristics on mortality is controversial [27]. In our study, no correlation was found between membrane transport characteristics and mortality.

In the literature, peritonitis history was shown to be an independent risk factor for all-cause mortality [9,17,23]. Similarly, we found an increased risk of mortality in peritonitis group compared to the nonperitonitis group. There was no difference between the peritonitis group and the nonperitonitis group in terms of patient survival in our study, similar to what was reported by Lasfar et al [8]. The International Society for Peritoneal Dialysis Guidelines considers the peritonitis rate of 0.67 per year to be acceptable [8,16]. But the incidence of peritonitis varies greatly from unit to unit. The reported peritonitis rates were 0.16–0.44 per patient year [8,18,23]. Similarly, this rate was 0.18 (0–14.9) attacks per patient year in our study. Indeed, this peritonitis rate in our centre (one every episode 80.6 patient-months) was higher than those reported in previous studies. In some studies, the overall peritonitis rate was 1 episode per 25–47.3 patient-months [6,8,19]. This may be due to differences in personal hygiene and self-care between patients and the education strategy of the center.

Technique failure rates were reported 31%–38.9% in previous studies [6,14,19]. This rate was lower in our study (26.4%). In other studies, different technique survival rates such as 87.9%–97.9% and 60.4%–84.5% were reported in the –1 and –3 years, respectively [8,14,17]. Our technique survival rates (92.6%, 81.8%, for –1 and –3 years, respectively) were similar to these studies. The reported mean technique survival was (61.7 ± 5.2 months) very close to our results (60 ± 55.2 months) [17]. PD-related infections are reported to be the most important factor for technique failure in PD patients [3,5,8–10,15,17,24]. In our series too, the most common cause of technique failure was infections. It causes patients to permanently transfer from PD to HD [15]. As reported in one study, technique failure had no effect on the mortality of our PD patients [10].

Rates of survival across our patients were similar to those observed in other studies (92.2% to 96.1% vs. 93.2%, 74.2% to 91.4% vs. 81.6%, and 57% to 89.3% vs %68.1, –1, –3, and –5 years, respectively) [8,14,19]. Compared with another study from Turkey, the mean survival rates of our elderly PD patients were 78.8% vs. 75.6% and 50.9% vs. 52.4% at 1 and 3 years, respectively [17]. The mean survival time of our elderly PD patients was similar to a study by Sakacı et al (44.4 vs 38.9 months) [17]. 

The majority of reports agree that patient survival is lower in diabetics compared to nondiabetics [8,18,19]. In this study, the survival was inferior and mortality was higher in diabetics compared to nondiabetics. As also seen in other studies, the survival was inferior and mortality was higher in the group aged ≥65 years compared to the 2 groups (aged <40 years and ≥40 years and <65 years) in our study [8,19]. 

In summary, in this study we found that, advanced age, the presence of diabetes mellitus, malignancy, and refractory heart failure increased mortality as independent risk factors. Diabetic patients, patients with comorbidity, and patients older than 65 years were the most important factors associated with mortality and patient survival. Although peritonitis increased the risk of mortality in our patients, it seemed to have no significant effect on patient survival. 

In conclusion, when beginning PD, patients with advanced age and presence of comorbidity (especially who have diabetes, malignancy, and refractory heart failure) should be marked for close follow up in order to have better long-term outcomes. Although peritonitis is the most feared complication of PD, our study showed that peritonitis did not reduce patient survival. The strengths of this study include its larger sample size and long follow-up time. This is the first study to define 9-year mortality predictors in PD patients in our centre. 

## Acknowledgments

We thank Sultan Erdoğan, peritoneal dialysis nurse, for her contribution of the study. There is no grant or other funding in this trial. This study was approved by Başkent University Institutional Review Board (Project no: KA19/196) and was supported by Başkent University Research Fund.

## References

[ref0] (2015). A global overview of the impact of peritoneal dialysis first or favored policies: an Opinion. Peritoneal Dialysis International.

[ref1] (2005). ESRD patients in 2004: global overview of patient numbers, treatment modalities and associated trends. Nephrology Dialysis Transplantation.

[ref2] (2001). Do the Y-set and double-bag systems reduce the incidence of CAPD peritonitis? A systematic review of randomized controlled trials. Nephrology Dialysis Transplantation.

[ref3] (2016). Mechanical complications of continuous ambulatory peritoneal dialysis: experience at the Ibn Sina University Hospital. Saudi Journal of Kidney Diseases and Transplantation.

[ref4] (2016). Impact of dialysis modality on technique survival in end-stage renal disease patients. Korean Journal of Internal Medicine.

[ref5] (2016). Recent analysis of status and outcomes of peritoneal dialysis in the Tokai area of Japan: the second report of the Tokai peritoneal dialysis registry. Clinical and Experimental Nephrology.

[ref6] (2018). Association between timing of peritoneal dialysis initiation and mortality in end-stage renal disease. Chronic Diseases and Translational Medicine.

[ref7] (2019). Long-term clinical outcomes of peritoneal dialysis patients: 10-year experience of a single unit from Tunisia. Saudi Journal Kidney Disease Transplantation.

[ref8] (2014). 9. Vikrant S. Long-term clinical outcomes of peritoneal dialysis patients: 9-year experience of a single center from north India. Peritoneal Dialysis International.

[ref9] (2009). Timing, causes, predictors and prognosis of switching from peritoneal dialysis to hemodialysis: a prospective study. BMC Nephrology.

[ref10] (2013). Peritoneal dialysis in rural Australia. BMC Nephrology.

[ref11] (Registry 2017). Registry of the Nephrology, Dialysis and.

[ref12] (2008). Registry of the Nephrology, Dialysis and Transplantation in Turkey.

[ref13] (2019).

[ref14] (2016). ISPD Peritonitis Recommendations: 2016 Update on Prevention and Treatment. Peritoneal Dialysis International.

[ref15] (2011). ISPD position statement on reducing the risks of peritoneal dialysis-related infections. Peritoneal Dialysis International.

[ref16] (2015). Clinical outcomes and mortality in elderly peritoneal dialysis patients. Clinics (Sao Paulo).

[ref17] (2019). Predictors of outcomes in patients on peritoneal dialysis: A 2-year nationwide cohort study. Scientific Reports.

[ref18] (2014). [Results of the cooperative study of Spanish peritoneal dialysis registries: analysis of 12 years of follow-up]. Nefrologia.

[ref19] (2019). Peritoneal dialysis as therapeutic option in heart failure patients. ESC Heart Failure.

[ref20] (1999). Peritoneal dialysis-associated peritonitis in Scotland (. Nephrology Dialysis Transplantation.

[ref21] (2015). Peritoneal dialysis: update on patient survival. Clinical Nephrology.

[ref22] (2017). The impact of peritoneal dialysis-related peritonitis on mortality in peritoneal dialysis patients. BMC Nephrology.

[ref23] (2006). Peritoneal dialysis in the US: evaluation of outcomes in contemporary cohorts. Kidney International Supplementary.

[ref24] (2019). Development of a risk prediction model for infection-related mortality in patients undergoing peritoneal dialysis. PLoS One.

[ref25] (2018). Prevalence of dynapenic obesity and sarcopenic obesity and their associations with cardiovascular disease risk factors in peritoneal dialysis patients. Kidney Research and Clinical Practice.

